# Noncontrast computed tomography factors predictive of extracorporeal shock wave lithotripsy outcomes in patients with pancreatic duct stones

**DOI:** 10.1007/s00261-018-1639-4

**Published:** 2018-05-15

**Authors:** Ri Liu, Weiwei Su, Jing Gong, Yu Zhang, Jianping Lu

**Affiliations:** 1 Department of Radiology, Changhai Hospital Affiliated to the Second Military Medical University, Changhai Road 168, Yangpu District, Shanghai, 200433 People’s Republic of China; 2 Department of Nuclear Medicine, Changhai Hospital Affiliated to the Second Military Medical University, Changhai Road 168, Yangpu District, Shanghai, 200433 People’s Republic of China

**Keywords:** Chronic pancreatitis, Pancreatic stones, Attenuation values, X-ray computed tomography, Extracorporeal shock wave lithotripsy

## Abstract

**Purpose:**

To assess the usefulness of factors unique to NCCT for the prediction of ESWL outcomes in patients with pancreatic duct stones.

**Materials and methods:**

We retrospectively evaluated 148 patients with multiple PDS who had undergone ESWL therapy. All patients received an examination for NCCT both before and after ESWL. The following parameters were measured and recorded: patient characteristics including sex and age; NCCT parameters including mean stone length, mean stone volumes before and after ESWL, mean value of CT attenuation, standard deviation of CT attenuation, variation coefficient of CT attenuation, skin-to-stone distance, and pancreatic duct diameter; ESWL outcome indexes including stone clearance rate calculated using the formula $\frac{V0 - V1}{V0} \times 100\%$, and the number of ESWL sessions. All patients were divided into groups based on their SCR: A group (SCR ≥ 90%), B group (SCR between 50% and 90%), and C group (SCR < 50%). Analysis of variance was used among the three groups to evaluate the potential predictors of SCR, and a receiver-operating curve was established to determine the optimal cutoff value.

**Results:**

ANOVA analysis revealed that MSD was the only significant predictor for SCR (*p* < 0.05), and ROC indicated an optimal cutoff value of +1000.45 HU, with a sensitivity up to 78.0% and specificity of 48.6%. Stones with MSD lower than +1000.45 HU had higher SCR (69.3%) than that of higher-density ones (59.6%). Pearson correlation analysis and histogram indicated a significant positive correlation between ESWL No. and MSL (*r* = 0.536), MSD (*r* = 0.250), SDSD (*r* = 0.247), and PDD (*r* = 0.227), all values being *p* < 0.01.

**Conclusion:**

MSD is the optimal predictor of ESWL efficacy, and PDS with lower MSD had a better clearance rate with fewer fragmentation sessions.

Extracorporeal shock wave lithotripsy (ESWL) was introduced to the clinic by Chaussy in 1980 [1] and was first applied to treat pancreatic duct stones (PDS) in 1987 [2]. This therapy has now been developed into the most common and widely accepted treatment option for PDS because of such advantages as safety, relative noninvasiveness, and high efficiency, with a reported success rate ranging from 46% to 91% in previous studies [3, 4]. However, this therapy may cause complications such as hemorrhage and infection, and its failure can not only make further auxiliary treatment more difficult but also increase physical pain for a patient as well as medical costs. It is therefore crucial that we complete a pretreatment investigation of stone characteristics most predictive of ESWL outcomes to devise an optimal treatment strategy for each PDS case.

A variety of factors can influence the outcome of ESWL, including patient features such as abdominal fat distribution and BMI [3, 5–7], ESWL shock frequency, and intensity [8, 9], as well as stone characteristics, for instance, location, size, composition, and fragility [10–12]. In recent years, Noncontrast Computed Tomography (NCCT) has become the preferred diagnostic tool for identification of abdominal stones with accuracy up to 96%–97% (sensitivity 95%; specificity 98%) [13]. Furthermore, this noninvasive imaging modality is being used more extensively in predicting stone fragmentation outcomes after ESWL. For example, it is known that the calculus fragility, which is in turn determined by stone composition, can affect ESWL efficacy. Further studies have revealed that variation in stone composition can be recognized as a difference in density on NCCT; although attenuation values are subject to several confounding factors, it is possible to detect subtle differences as low as 0.5%, thus making this parameter capable useful for measuring stone fragility and determining ESWL outcomes [14, 15]. Gupta et al. [16] also reported that urinary calculi with a diameter > 1.1 cm suggested a poor ESWL outcome. Perks et al. [17] found a favorable ESWL efficacy in the treatment of urinary stones with a skin-to-stone distance < 9 cm.

Although there are currently a variety of reports about the use of NCCT in prediction of ESWL outcomes for urinary calculi, to our knowledge, corresponding studies on PDS are rare and not all-inclusive. One reason may be that pancreatic stones have a relatively lower incidence than calculi of the urinary or biliary system. Another factor may be the limited ESWL technical knowledge in most hospitals, which leads PDS patients to seek services elsewhere, and thus, hospitals are unable to obtain a sufficient number of patients for scientific research.

The present study collected data from Chang Hai Hospital which has one of the largest Digestive Endoscopy Centers in the Asia–Pacific region and the only one that performs pancreatic ESWL (*P*-ESWL) in China. Due to these factors, we were able to recruit a large number of PDS patients. Our aim was to analyze stone characteristics obtained from NCCT images and find positive pretreatment predictors for ESWL efficacy in terms of stone clearance rate (SCR) and the number of ESWL sessions (ESWL No.). Determination of these predictors would ultimately help in making the optimal treatment decision, avoiding unnecessary ESWL radiation exposure, and minimizing additional physical suffering and financial burden.

## Materials and methods

### Patients population

Between Nov 2014 and Nov 2016, we conducted a retrospective review of 148 clinical data from patients with diagnosed chronic pancreatitis (CP), all of them with multiple PDS larger than 5 mm in the pancreatic head region. Patients who had undergone pancreatic surgery before ESWL or failed to provide ascertained fragment outcome were excluded from this study.

The main clinical symptom for all patients was abdominal pain for which they underwent formal ESWL therapy. NCCT examination was performed before and after treatment. All clinical data were completely detailed, and this study was approved by the Institutional Review Board, and each patient provided informed consent.

### Extracorporeal shock wave lithotripsy

All patients underwent the regular and successive ESWL therapy sessions until the largest stone was fragmented to 3 mm or less without any complication. Pretreatment with intravenous anesthesia (flurbiprofen and remifentanil) was necessary to alleviate pain, and subjects were instructed to lie on their backs with their bent knees held above their hips. Two experienced gastroenterologists performed the ESWL.

Lithotripsy was conducted using a third-generation electromagnetic lithotripter (Compact Delta II; Dornier Med Tech, Wessling, Germany). All radiopaque stones were detected with the help of a fluoroscope, and radiolucent stones were found after insertion of a nasopancreatic tube secondary to pancreatic sphincterotomy to aid in targeting during ESWL. The lithotripsy could last for several days with repeated ESWL sessions, and each session lasted between 60 and 90 min, with a frequency of 100 shocks per minute and an intensity of 6 (16,000 kV) on a scale of 1 to 6. The numbers of ESWL sessions were accurately recorded for each patient.

### Stone characteristics as determined by NCCT

NCCT examination was performed using a 320-detector CT scanner (Brilliance-320; Toshiba, Japan). During scanning, each patient was instructed to hold their breath at the end of inspiration and remain in the supine position. CT scanning parameters were as follows: tube voltage: 120 kV; tube current: 500 mA; collimation: 128 × 0.625 mm; gantry rotation time: 0.5 s; beam pitch: 0.915; reconstruction thickness: 1 mm; and reconstruction interval: 1 mm. The scanning field ranged from the superior border of the liver to the anterior superior iliac spine; all images were reconstructed using a standard algorithm within the bone window.

**Fig. 1:**
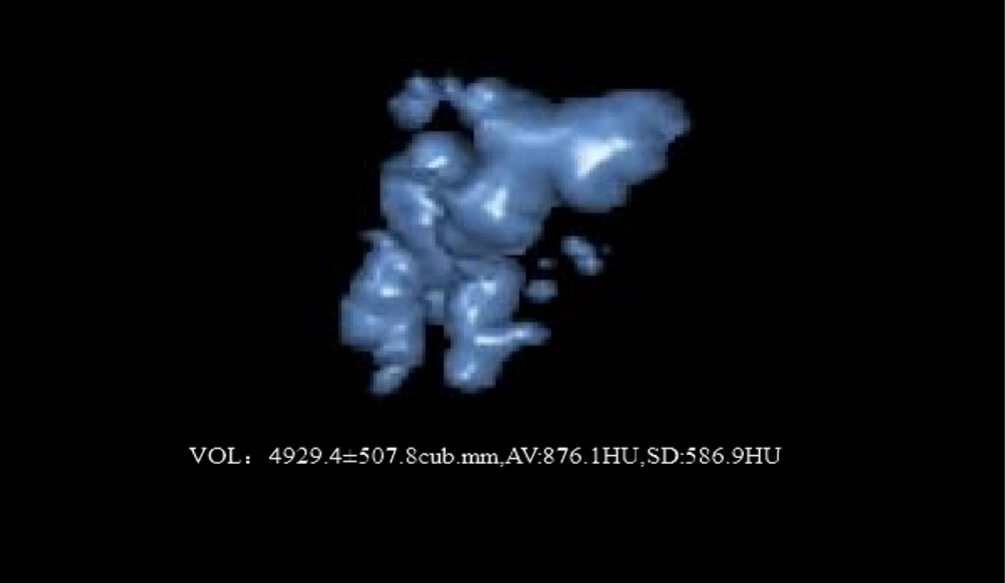
The Philips IntelliSpace Portal software automatically calculated MSD (876.1HU), SDSD (586.9HU), and volume (4929.4 mm^3^)

**Fig. 2:**
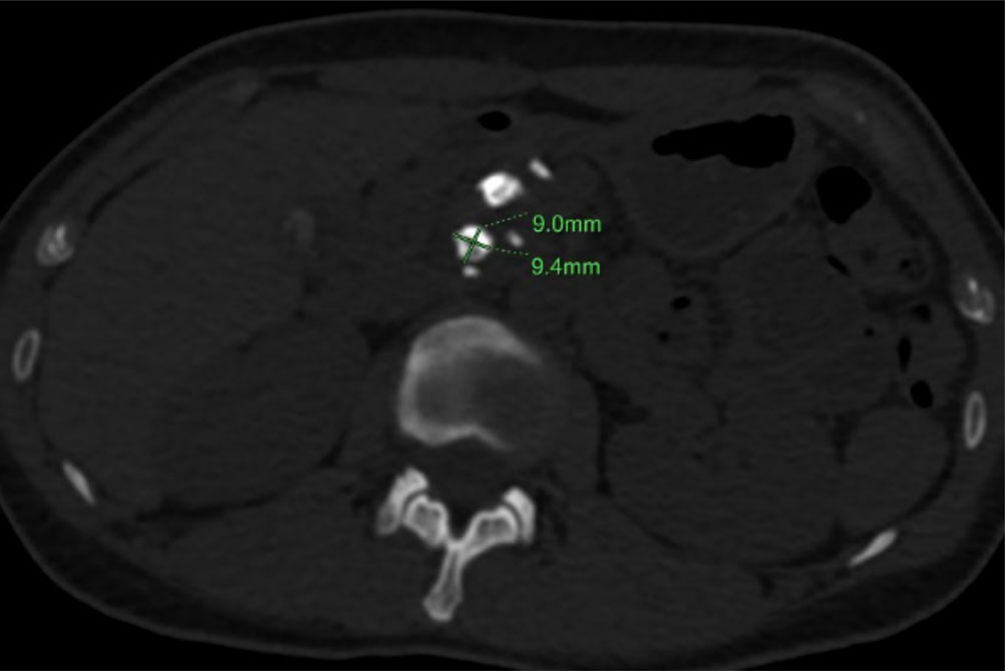
MSL was obtained by manual measurement and calculating the mean value of maximum transverse length (*a* = 9.4 mm) and the shortest diameter (*b* = 9.0 mm) on the biggest stone that was 9.2 mm

**Fig. 3:**
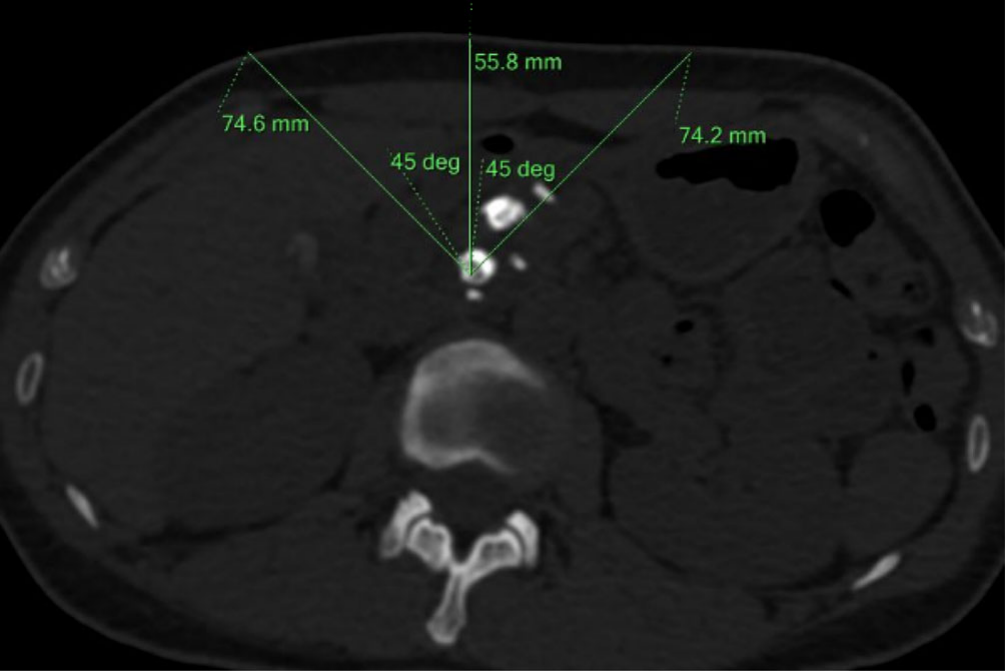
SSD was obtained by calculating the mean distance from the stone center to the skin surface at 0° (55.8 mm), 45° (74.6 mm), and − 45° (74.2 mm) on the axial NCCT image that was 68.2 mm

All thin slice NCCT images were imported into a software called Philips IntelliSpace Portal version 6.0.4 (Netherlands) which automatically recognizes all PDS and outputs indexes including preoperative stone volume (V0) and postoperative stone volume (V1), mean value of CT attenuation (MSD), and standard deviation of CT attenuation (SDSD) (Fig. 1). The variation coefficient of CT attenuation (VCSD) value was set when SDSD was divided by MSD. Subsequently, we located the biggest stone with the maximum transverse length (a) and measured the shortest diameter (b) at the same scanning slice. The mean stone length (MST) was calculated using the formula: MST = (a + b)/2 (Fig. 2). Pancreatic duct diameter (PDD) was determined by measuring the diameter of the dilated position along the obstructed pancreatic duct distally to the pancreatic head. Finally, we determined the value of skin-to-stone distance (SSD) by measuring and calculating the average vertical distance from the center of the biggest stone to the skin surface at 0°, 45°, and −45° (Fig. 3), according to the theory proposed by Pareek [7] et al. Final SCR was calculated with the formula: ${\text{SCR}} = \frac{V0 - V1}{V0} \times 100\%$.

### Statistical analysis

We used SPSS software (version 21.0, Chicago, IL) for statistical analysis. All continuous variables are presented as a mean (SD) and categorical variables as a percent. Potential factors influencing SCR by ESWL were determined by analysis of variance (ANOVA). The receiver-operating characteristic (ROC) curve was plotted to find the optimal cutoff value with maximal sensitivity and specificity, and area under the curve (AUC) was calculated to illustrate the value’s predictive power for SCR. In order to identify factors associated with ESWL No., we performed Pearson correlation analysis and constructed the relevant histograms. A *p* value less than 0.05 was considered indicate a significant difference or correlation.

## Results

### Patients and ESWL outcomes

**Fig. 4:**
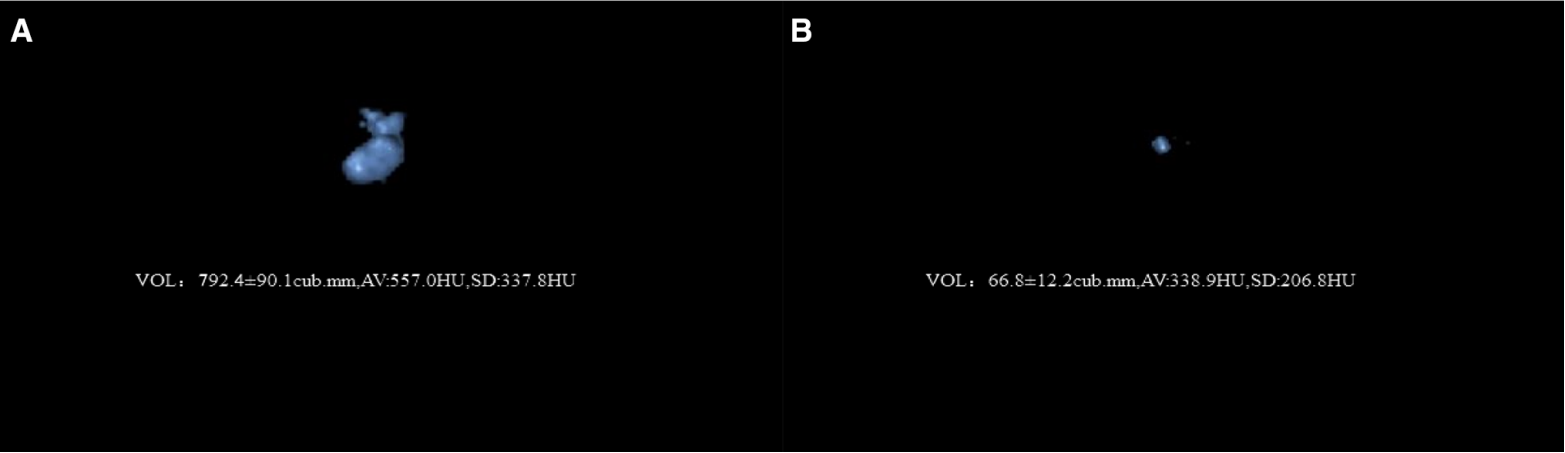
A 21-year-old young man with multiple PDS (**A**; V_0_:792.4 mm^3^) was hospitalized in the digestive department and underwent the formal and consecutive ESWL therapy. The NCCT scanning images were processed by the Philips IntelliSpace Portal software and the computed stone parameters were as follows: MSL: 10 mm; MSD: 557HU; SDSD: 337.8HU; VCSD: 0.6; SSD: 141.2 mm; PDD: 2 mm. The therapeutic outcome was optimistic (**B**) after 5 sessions of ESWL with a high SCR up to 91.6% (V_1_:66.8 mm^3^). The patient’s primary clinical symptom related to pancreatitis was greatly improved

In the end, 148 patients were included in this study, including 107 men and 41 women with the mean age of 44.8 ± 13.4 years (range, 12 to 75 years). All subjects had multiple PDS in the pancreatic head region with a mean primary volume of 5197.4 (5871.9) mm^3^. Additionally, all had undergone formal ESWL therapy for a collective total of 356 sessions (mean 2.4 per patient; range 1–8; 37 cases of 1 session; 61 cases of 2 sessions; 19 cases of 3 sessions; 20 cases of 4 sessions; 8 cases of 5 sessions; 2 cases of 6 sessions and 1 case of 8 session) (Fig. 4a, b).

The mean volume of residual stones was 1771.5 (2560.0) mm^3^. Consequently, the calculated mean SCR was 63.6 ± 23.7%, with a range from 0.3% to 100%. Specifically, 28 subjects, including 17 men and 11 women, were defined as A group with an average SCR of 94.2% (range: 90% to 100%); 79 subjects (56 men and 23 women) were defined as B group with an average SCR of 68.4% (range: 50.6% to 88.4%); and C group included 41 subjects (33 men and 8 women) with an average SCR of 33.3% (range: 0.3% to 49.9%).

Other basic information about patient characteristics, NCCT parameters, and ESWL outcome indexes are shown in Table 1.

**Table 1:** Baseline information about patients’ characteristics, the NCCT parameters and ESWL outcome (*n* = 148)

Variable	Value
Patient characteristics	
Age, mean (SD), years	44.8 (13.4)
Male sex, *n* (%)	107 (72.3)
NCCT parameters	
Mean stone length (MSL), mean (SD), mm	9.5 (4.9)
Mean stone density (MSD), mean (SD), HU	1181.8 (479.7)
Standard deviation of stone density (SDSD), mean (SD), HU	309.7 (135.5)
Variation coefficient of stone density (VCSD), mean (SD), 100%	0.3 (0.2)
Skin-to-stone distance (SSD), mean (SD), mm	91.5 (19.5)
Pancreatic duct diameter (PDD), mean (SD), mm	9.3 (4.0)
Stone volume before surgery (V0), mean (SD), mm^3^	5197.4 (5871.9)
Stone volume after surgery (V1), mean(SD), mm^3^	1771.5 (2560.0)
ESWL outcome	
Stone clearance rate (SCR), mean, %	63.6 (23.7)
Number of ESWL sessions (ESWL No.), mean (SD), 1	2.4 (1.3)

*Note* Data are presented as mean (standard deviation) or percentage.

### NCCT predictive factors for SCR by ESWL

**Fig. 5:**
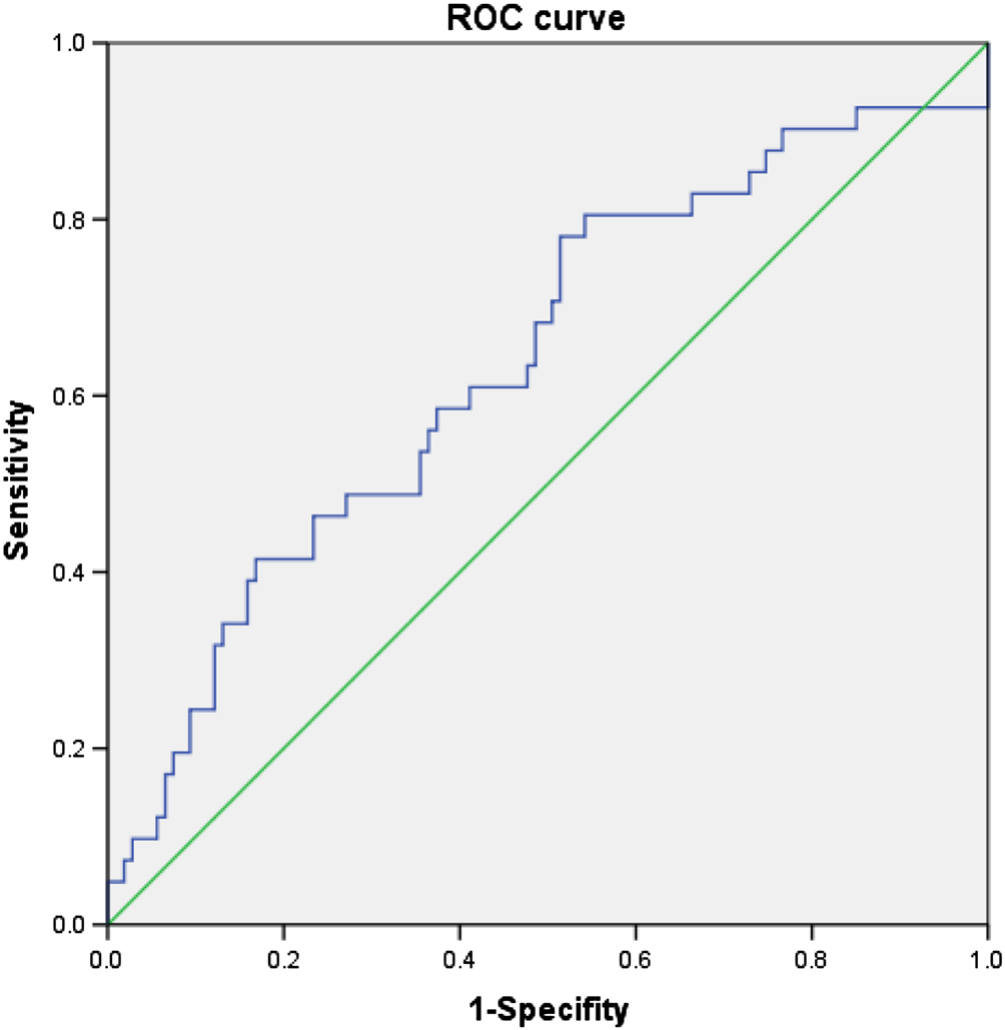
Receiver-operating curve of MSD for predicting SCR of ESWL

In order to identify predictors for SCR by ESWL, analysis of variance (ANOVA) was performed. We found that MSD was a unique predictor for SCR in this study with a significant difference among the three groups (*p* < 0.05) as shown in Table 2. A ROC curve was further plotted (Fig. 5), and the cutoff value was determined to be 1000.45HU with a corresponding sensitivity up to 78% and a specificity of 48.6%. A relatively high AUC value (0.6373) revealed the predictive power of MSD for SCR. We also found that 61 patients with MSD<1000.45 had a relatively higher SCR averaged 69.3% than those with higher-density stones (SCR, 59.6%).

**Table 2:** Comparison of quantitative parameters on NCCT for PDS among three groups

	A group	B group	C group	*p* value		
				AB	AC	BC
MSL (mm)	9.4 ± 4.6	9.9 ± 5.2	8.7 ± 4.3	0.628	0.56	0.196
MSD (mm)	1069.4 ± 432.1	1141.8 ± 439.7	1335.6 ± 553.4	0.487	0.023*	0.035*
SDSD (mm)	328.5 ± 132.6	298.0 ± 132.3	319.6 ± 144.4	0.309	0.788	0.411
VCSD (%)	0.3 ± 0.2	0.3 ± 0.1	0.3 ± 0.1	0.098	0.117	0.916
SSD (mm)	86.9 ± 25.9	92.2 ± 17.0	93.3 ± 18.9	0.219	0.187	0.782
PDD (mm)	9.3 ± 4.3	9.4 ± 3.2	8.9 ± 5.1	0.862	0.742	0.537

*Note* Date are mean ± standard deviation

*p < 0.05; analysis of viariance (ANOVA)

### NCCT predictive factors for ESWL sessions

Pearson correlation analysis revealed a significantly positive correlation between ESWL No. and PDS indexes including MSL (*r* = 0.536, *p* < 0.01), MSD (*r* = 0.250, *p* < 0.01), SDSD (*r* = 0.247, *p* < 0.01) and PDD (*r* = 0.227, *p* < 0.01) (Table 3). The mean values of MSL, MSD, SDSD, and PDD were greater in patients with ESWL No. > 2.4 sessions than those with fewer ESWL sessions (12.6 vs 7.9; 1341.vs 1100.3; 361.7 vs 283.2; 10.3 vs 8.8).

**Table 3:** Correlation between PDS parameters on NCCT and ESWL No.

	ESWL No.	
	*r*	*p*
MSL (mm)	0.536**	0.000
MSD (mm)	0.250**	0.002
SDSD (mm)	0.247**	0.002
VCSD (%)	0.021	0.795
SSD (mm)	−0.057	0.491
PDD (mm)	0.227^**^	0.005

**p < 0.01; the Pearson correlation analysis

## Discussion

Chronic pancreatitis (CP) is an intractable and progressive inflammatory disease often complicated by pancreatic duct stones (PDS). These stones are the primary contributor to the abdominal pain of CP patients because they cause parenchymal or functional damage to the pancreas. ESWL is currently the most common nonsurgical intervention for PDS patients, especially for those with multiple, large stones that cannot be completely removed with endoscopic retrograde cholangiopancreatography (ERCP). The success rate of ESWL alone can reach up to 100% according to some reports [18, 19].

In the current study, we identified two indexes that can be used to predict ESWL outcomes. One of these indexes was stone clearance rate (SCR), which was calculated based on the percentage decrease in stone volume after ESWL therapy; another was ESWL No., both of which were recorded during the course of therapy. We comprehensively described the effect of quantitative stone parameters on NCCT and analyzed their correlation with the ESWL outcomes. Our major findings were concluded from these analyses.

Presently there are a variety of reports on the density of solitary stones in the urinary system and it is widely accepted that CT density is a representation of stone hardness, determined by stone composition, which can further affect ESWL efficacy. However, corresponding studies about solitary PDS are rare, and there are no reports about multiple PDS that predicting ESWL efficiency using NCCT. In previous studies about the ureteral calculi, both MSD and SDSD were acknowledged to be independent predictors for ESWL efficacy because they correlate with stone hardness and stone fragility, respectively [20]. In a study by Hiroshi et al., lower stone density had a significant association with complete stone removal [21]. Similarly, in the present study software was used to automatically compute the MSD and SDSD to represent the mean density level of multiple PDS.

Results of this analysis revealed that MSD and SDSD had the approximately equal correlation coefficients with ESWL No. (MSD *r* = 0.250; SDSD *r* = 0.247), indicating a comparable role in predicting the required number of ESWL sessions for total stone clearance. Importantly, we were pleased to find a significant difference in MSD among the three SCR groups with *p* < 0.05. Further ROC analysis showed that the cutoff value of MSD is 1000.45 HU with a maximal sensitivity up to 78% and specificity of 48.6%. Moreover, 61 patients with an MSD smaller than 1000.45 HU had a relatively higher SCR over 69.3% and smaller ESWL No., usually less than 2 sessions, compared with higher-density stones; these results corresponded with those of previous study [1]. Taken together, we reached the conclusion that MSD is the optimal predictor for ESWL outcomes in terms of both SCR and ESWL session number. This result may help us to better screen for the most suitable patients for ESWL treatment.

*ESWL alone = MSL* In order to illustrate PDS size, we calculated the mean maximum transverse length and the shortest diameter of the biggest stone to minimize bias caused by the irregularity of stone shape. A study by Lapp et al. [22] demonstrated technical success (TS) of ESWL for PDS with a diameter less than 5 mm on pancreatogram imaging and we also found that PDS with a diameter larger than 12 mm were predictive of unsuccessful TS. However, in the present study, we found that PDS size did not affect SCR but we detected a significant positive correlation between MSL and ESWL No. (*r* = 0.536**) according to Pearson analysis. This correlation was evident due to the rising trend in the histogram with increasing PDS size. Patients with ESWL No. greater than 2 sessions had a larger stone MSL with average 12.6 mm compared to those who only required one or two sessions of ESWL with PDS on average of 7.9 m,. In other words, larger stones require more ESWL sessions for successful fragmentation to sizes less than 3 mm, but this does not necessarily indicate a higher SCR. In clinical practice, we should inform patients about psychological preparation for many ESWL treatments for large pancreatic stones.

*ESWL alone = PDD* During the formation of pancreatic stones, it is assumed that a damaged pancreatic duct (PD) is constricted which causes pancreatic fluid congestion and further exacerbates local obstruction and distal dilation of the duct which can stimulate the calculus forming. In a study done by Lapp et al. [22], CP patients with a dilated duct (> 8 mm) had more chances of ESWL failure. However, other results dispute the influence of PD stricture on the ESWL clearance [23]. In the current study, we didn’t find any significant difference of PDD between SCR groups. One possible interference factor, if any, is our measurement strategy, which was limited by the resolution of NCCT making it difficult to precisely locate the measuring position for the dilated duct. This measurement should be performed on ERCP or magnetic resonance (MR) images.

Moreover, most previous studies were based on treatment of a solitary stone in a dilated main pancreatic duct (MPD), but all patients in this study had multiple stones in both MPD and in branches of the dilated pancreatic duct (BPD). The PDD value may not effectively represent the overall level of all pancreatic ducts. Nevertheless, the positive correlation between PDD and ESWL No. (*r* = 0.227**, *p* < 0.01) indicated that more severe dilation of the distal PD necessitates more sessions of ESWL. This finding can help clinicians estimate the feasibility and necessity of ESWL in wise treatment decisions.

*no sense = SSD* The parameter SSD as a fragment predictor for ESWL was first introduced by Pareek et al. in a 2005 study of patients with lower pole renal stones [24]. It was concluded that SSD can respond to stone localization, amount of subcutaneous as well as visceral fat tissue, and renal parenchymal thickness [25]. However, the role of SSD as an ESWL predictor still remains controversial. Perks et al. [17], Cho et al. [26], Wiesenthal et al. [27], and Lee et al. [12] have found a SSD threshold of 9 cm, 10 cm, 11 cm, 11.43 cm respectively for differentiating ESWL outcomes, and SCR decreased as the length increased. However, several other published studies [26, 28, 29] were of the opposite opinion due to failure to identify such a positive correlation.

Cho et al. [26] analyzed two possible factors that may be the cause of the discordance among the previous studies. One of the reasons was patient race, as the researchers concluded that the physical difference between Asian and Western patients can influence the effect of SSD on SCR. Additionally stone location was also argued in another study [30] to be predictive of SSD only for renal stones but not for ureter stones because of their longer SSD could lead to higher attenuation of ESWL shock power. Finally, in this study of PDS, we did not find a significant influence of SSD on either ESWL sessions or SCR. This may be due to the fact that ESWL of pancreatic calculi is performed on the anterior part of the abdomen, which is highly susceptible to deformation by squeezing of the sonotrode.

## Conclusion

By studying a sufficiently large number of patients with pancreatic duct stones who had undergone ESWL therapy, we were able to make a comprehensive analysis of potential predictors of ESWL outcomes and fully demonstrated the important value of quantitative NCCT in the assessment of need for and efficacy of ESWL. Nevertheless, more extensive studies including patient physical characteristics, previous clinical therapy, and follow-up data, which may be suggestive of a cure for patients suffering from chronic pancreatitis with PDS, are desired.
